# Reducing malaria misdiagnosis: the importance of correctly interpreting Paracheck Pf^® ^"faint test bands" in a low transmission area of Tanzania

**DOI:** 10.1186/1471-2334-11-308

**Published:** 2011-11-03

**Authors:** Lisa K Allen, Jennifer M Hatfield, Giselle DeVetten, Jeremy C Ho, Mange Manyama

**Affiliations:** 1 Faculty of Medicine, University of Calgary, 3330 Hospital Drive NW, Calgary AB, T2N 4N1, Canada; 2 Bugando University College of Health Sciences, Mwanza P.O. Box 1464, Tanzania

## Abstract

**Background:**

Although malaria rapid diagnostic tests (RDTs) have been extensively evaluated since their introduction in the early 1990's, sensitivity and specificity vary widely limiting successful integration into clinical practice. This paper reviews specific issues surrounding RDT use in field settings and presents results of research investigating how to interpret "faint test bands" on ParaCheck Pf^® ^in areas of low transmission in order to reduce malaria misdiagnosis.

**Methods:**

A multi-phase cross-sectional study was conducted at a remote hospital in the northern Tanzanian highlands. Capillary blood samples were taken from consenting participants (n = 319) for blood smear and ParaCheck Pf^® ^testing. Primary outcome variables were sensitivity, specificity and proportion misdiagnosed by ParaCheck Pf^® ^and local microscopy. ParaCheck Pf^® ^"faint bands" were classified as both true positives or true negatives during evaluation to determine appropriate clinical interpretation. Multivariate logistic regression adjusted for age and gender was conducted to determine odds of misdiagnosis for local microscopy and ParaCheck Pf^®^.

**Results:**

Overall, 23.71% of all ParaCheck Pf^® ^tests resulted in a "faint band" and 94.20% corresponded with true negatives. When ParaCheck Pf^® ^"faint bands" were classified as positive, specificity was 75.5% (95% CI = 70.3% - 80.6%) as compared to 98.9% (95% CI = 97.0% - 99.8%) when classified as negative. The odds of misdiagnosis by local microscopy for those > 5 years as compared to those ≤ 5 years are 0.370 (95% CI = 0.1733 - 0.7915, p = 0.010). In contrast, even when ParaCheck Pf^® ^faint bands are considered positive, the odds of misdiagnosis by ParaCheck Pf^® ^for those > 5 years as compared to those ≤ 5 years are 0.837 (95% CI = 0.459 - 1.547, p = 0.5383).

**Conclusions:**

We provide compelling evidence that in areas of low transmission, "faint bands" should be considered a negative test when used to inform clinical decision-making. Correct interpretation of RDT test bands in a clinical setting plays a central role in successful malaria surveillance, appropriate patient management and most importantly reducing misdiagnosis.

## Background

Current recommendations of effective, yet expensive artemisinin-based combination therapies (ACT) for malaria in Sub-Saharan Africa have increased the importance of laboratory-confirmed diagnosis [1-3]. The current diagnostic "gold standard" for malaria diagnosis is direct microscopic visualization of parasites on thick and/or thin blood smears [4,5]. Unfortunately, in many health facilities in Sub-Saharan Africa there is a lack of properly functioning microscopes, quality control systems and well-trained laboratory technicians [6]. Due in part to these limitations, clinicians at health facilities may develop limited trust in the accuracy of microscopy results and rely solely on clinical judgment even when laboratory results are available [7]. This is particularly troubling since the clinical symptoms of malaria are non-specific and overlap with a number of other tropical infectious diseases [8]. As a result, clinically similar diseases may be treated as malaria, reducing specificity, promoting over-use of anti-malarial drugs and reducing the quality of care for patients [9].

Rapid Diagnostic Tests (RDTs) for malaria have substantial potential to help address these issues, especially in resource poor areas [9]. In order for RDTs to be effective, they must have high sensitivity for *Plasmodium *species detection, paired with high specificity necessary to successfully reduce malaria associated morbidity and mortality and accurately diagnose other tropical diseases [10]. This successful combination will aid in the reduction of malaria misdiagnosis, decrease treatment costs, reduce misperceptions of therapeutic failures when fever is caused by other diseases, and reduce selection pressure that leads to the development of drug resistance [11].

However, RDTs are not a perfect solution and there is growing concern regarding their effectiveness, quality, accuracy and perceived value. This paper reviews some of the current literature on challenges associated with RDTs and presents the results of research investigating the interpretation of "faint bands" which are a problematic feature of some malaria RDTs [3,11,12].

### Current Challenges with Malaria RDTs

Since the introduction of RDTs in the early 1990's, there have been over 100 published trial reports [13]. One of the first and most extensively tested and utilized RDTs, ParaCheck Pf^® ^detects histidine rich protein 2 (HRP2) produced uniquely by *Plasmodium falciparum *[12,14-21]. Although the sensitivity of ParaCheck Pf^® ^is consistently reported to be above 90% in hospital and field based studies, specificity varies from as low as 52% up to 99.5% [11]. Variability in specificity may be associated with the quality of the reference standard used, as studies rely on a variety of tests (single or dual field microscopy, single or dual hospital microscopy and polymerase chain reaction (PCR)) [11]. Beyond this limitation, it has also been suggested that ParaCheck Pf^® ^false positives can be attributed to: reading the test after fifteen minutes, application of too much blood, inappropriate dilution/concentration of buffer, contamination of the sample, backflow of sample and conjugate leading to entrapment at the detection line, high titers of non-specific anti-bodies due to viral infection or the presence of rheumatoid factor [2,6,14,22]. The appearance of "faint test bands" on RDTs such as ParaCheck Pf^® ^also may have implications for producing false positives during practical interpretation.

In previous reports from a hyper-endemic area of Uganda, Kyabayinze et al. (2008) identified "faint test bands" in ParaCheck Pf^® ^(bands that can only be seen in good light) and classified them true positives using concordance values between two HRP2 RDTs [5]. Aside from this study, the majority of previous research either does not identify faint bands, or may have included them as either true positives or negatives without further investigation. As malaria prevalence continues to fall due to successful preventative measures, and if RDTs are truly the answer to improve the accuracy of malaria diagnosis, it is imperative to have clear guidelines for interpretation of ParaCheck Pf^® ^results in various malaria transmission settings.

In this paper, we evaluate the sensitivity and specificity of ParaCheck Pf^® ^against microscopy in an area of low malaria transmission in the Tanzanian highlands and determine whether, in this setting "faint bands" are most frequently associated with true positives or negatives. In addition, we provide a comparison of malaria misdiagnosis based on local microscopy as compared to ParaCheck Pf^® ^and use these findings to propose potential implications for areas of low malaria transmission.

## Methods

### Study site

This study was conducted at a rural hospital in the Ngorongoro Conservation Area (NCA) located within the highlands of northern Tanzania where the temperature can range from 2°C to 35°C annually. The hospital laboratory relies on generator power for electricity and sunlight through open windows is the major source of lighting. Altitude in the NCA ranges from 1000 m to over 3000 m placing the majority of the population in a low transmission zone for malaria. Based on hospital outpatient records, the reported prevalence of malaria at the hospital was over 40%, but it is anticipated that the actual prevalence of malaria in the NCA is estimated to be 9.3% [7]. Interpretation of these statistics indicates that over-diagnosis of malaria may be of concern in this area.

### Study design

A multi-phase cross-sectional study was conducted to determine accuracy of ParaCheck Pf^® ^as compared to local hospital microscopy and external quality control (QC) performed at the Bugando University College of Health Sciences (BUCHS) in Mwanza, Tanzania. Study design was based on the World Health Organization (WHO) Evaluation of diagnostic tests for infectious diseases: general principles [23] and the WHO Evaluation of rapid diagnostic tests: malaria [24]. A demographic and health seeking behavior questionnaire was administered in parallel to participants in all study groups in order to determine if individual-specific factors are associated with ParaCheck Pf^® ^sensitivity, specificity and the appearance of faint bands (see Appendix A for questionnaire).

### Sample size calculation

Sample size was calculated using 95% confidence, an expected malaria prevalence of 9.3% [7] and precision level of 5%. The simple formula of n = Z^2 ^P(1-P)/d^2^, where n = sample size, Z = Z statistic for a level of confidence, P = expected prevalence or proportion and d = precision was used for calculations [25]. The resulting total sample size required was n = 129. This sample size was doubled in order for the age groups to be dichotomized during logistic regression analysis resulting in a final total sample size of n = 258.

### Participants

#### Inclusion criteria

patients presenting with clinical features of malaria sent for microscopy investigations after giving informed consent.

#### Exclusion criteria

any women with known or suspected pregnancy, patients with severe and/or complicated malaria, and patients with severe and complicated co-morbidities as defined by the clinical health officers. Patients were excluded if they had received anti-malarial therapy at the hospital in the last 2 weeks.

### Sampling strategy

Using convenience sampling [26], research personnel identified all potential participants presenting to the out-patient department of the hospital within the data collection time frame (January, May and June of 2009). Although a random sample of potential participants would be ideal, it has limited feasibility as an average of 10 - 15 patients/day present with malaria-like symptoms at the study site.

### Patient Demographics and Health Seeking Behaviors

Following provision of informed consent, patient demographics (age, gender, Maasai age class and previous cases of malaria) and health seeking behaviors (days waiting before presenting to hospital and time traveled) were recorded using a previously pilot tested and validated questionnaire (additional file 1). This enabled the researchers to investigate associations with positive, negative and misdiagnosed microscopy and ParaCheck Pf^®^test results.

### Microscopy

In the laboratory, blood was obtained by a finger prick and a thick and thin blood smear were prepared according to standard WHO procedures [27]. One of the laboratory technicians working in the hospital laboratory stained slides with either Fields stain or Giemsa and estimated parasite density against 200 white blood cells. At least 100 fields on each slide were examined before the slide could be declared negative for malaria parasites [27]. Local hospital microscopists were blinded to the results of ParaCheck Pf^® ^and to the clinical outcome [24]. Blood slides were stored in a cool and dark location until being sent to BUCHS to be read by 2 independent expert parasitologists. QC parasitologists were blinded to each other as well as the local hospital microscopy results and the ParaCheck Pf^® ^results.

All microscopists both at the rural hospital in the Ngorongoro and BUCHS hold a three-year diploma in laboratory techniques with more than six years working experience in the laboratory (parasitology section). In addition they all have been attending refresher courses to improve their skills in malaria microscopy. At both sites, the WHO standard operating procedures for microscopy were followed [27].

### ParaCheck Pf^®^

ParaCheck Pf^® ^(Orchid Biomedical Systems, Verna Goa, India) was used for the qualitative detection of *Plasmodium falciparum*. The tests were stored in a cool and dry location for the duration of the trial until use, and each package was examined for the integrity of the desiccant. Standard protocol was followed by trained research personnel, where 5 μl of fresh capillary blood was transferred using the provided loop applicator to the test window and then 6 drops of sample buffer were added to the buffer well. Once a ParaCheck Pf^® ^package was opened, it was used immediately. Trained research staff interpreted RDT results for exactly 15 minutes following the addition of the buffer solution as recommended by the manufacturer. In all cases, interpretation of ParaCheck Pf^® ^results was overseen and confirmed by the lead author. In order to ensure integrity of interpretation, all research personnel involved in ParaCheck Pf^® ^interpretation were blinded to both the results of microscopy as well as the clinical decision.

The presence of a band in both the control and test line indicates a positive test for *P. falciparum*. A negative result was indicated by the presence of a control band and the absence of a test band. A "faint band" was identified using the same definition as Kyabayinze et al. (2008), where "faint bands" are those that are only visible in good light. Good light is defined as viewing the ParaCheck Pf^® ^test in direct sunlight (all tests were conducted between 9 am and 4 pm). A test was considered invalid when the control line did not appear or the test failed to clear remaining blood from the test area. The final diagnosis and further patient management and treatment were solely based on microscopy results.

### Quality Control

#### Microscopy

After completion of standard WHO microscopy procedures [27], a blood smear was considered to be either a true positive or true negative only when both external QC readers agreed on the result. Percent agreement and kappa values were used for analysis [28]. Parasitemia level was not considered for true positives, only that both QC readers identified the slide as positive. Parasitemia was used as a measure of infection severity for clinical purposes but was not taken into account during QC measures.

#### ParaCheck Pf^®^

Timers were used to ensure that tests were read at exactly 15 minutes. In the case of a "faint band" a second observer was consulted to determine the presence of a "true faint band". "Faint bands" were considered present only if both observers agreed.

### Ethical considerations

Ethical approval from the Conjoint Health Ethics Board (CHREB) at the University of Calgary and the National Institute of Medical Research (NIMR) in Tanzania as well as a research permit required in Tanzania from the Commission on Science and Technology (COSTECH) were received.

### Data analysis

All data were entered into Microsoft Excel and checked by a second researcher for consistency against original data sources, then analysis was done using STATA version 11.0. The variable misdiagnosis was created for both local microscopy and ParaCheck Pf^® ^by coding the test result as a misdiagnosis when there was a disagreement with the external QC result and the test result (i.e. ParaCheck Pf^® ^was positive and the QC result was negative, or vice versa). Logistic regression adjusting for age and gender was conducted to determine the odds of misdiagnosis using ParaCheck Pf^® ^or local microscopy. All confidence intervals are calculated using the Exact-Method.

## Results

### Malaria Prevalence

For prevalence calculations, the population is defined as patients who are suspected of malaria by a clinical officer or physician at the outpatient department of the local rural hospital. The resulting prevalence estimates are shown in Table 1.

**Table 1:** The estimated prevalence of malaria in patients who are suspected of malaria by a clinical officer or physician at the out-patient department of the local rural hospital using local microscopy, ParaCheck Pf**^® ^**and quality control microscopy.

Diagnostic Method	Estimated Prevalence
Local Microscopy	46.4% (95% CI = 40.6%-52.2%)

ParaCheck Pf^®^ (If classify faint bands as -)	1.7% (95% CI = 0.2%-3.2%)

ParaCheck Pf^®^ (If classify faint bands as +)	25.4% (95% CI = 20.4%-30.5%)

Quality Control Microscopy	2.1% (95% CI = 0.4%-3.8%)

### Study Participants

A total of 319 study participants were divided into three study groups: 1) malaria-like symptoms ≤5 years (n = 98), 2) malaria-like symptoms > 5 years (n = 122) and 3) a control group presenting with non-malaria-like symptoms > 5 years (n = 71). Due to inconsistencies and missing data, the sample size used for analysis was n = 291. Of the 291 participants, there were approximately equal numbers of males and females (females n = 147), a larger proportion of patients were > 5 years (n = 193), waited longer than 1 day to present at the hospital (n = 225) and a small proportion had to travel longer than 3 hours to get to the hospital (n = 40).

### Quality Control

The external QC readers were regarded as the gold standard for this evaluation. Evaluation of external QC reader inter-rater reliability resulted in 97.25% agreement (kappa = 0.5859, SE = 0.0583, p < 0.0001). There were 8 cases out of 291 that the QC readers did not agree. These cases were then excluded from calculations that use true positives or true negatives. The proportion of true positives determined by agreement between both external QC readers was 2.10% (6/283).

### ParaCheck Pf^®^

Overall, 23.71% of all ParaCheck Pf^® ^tests resulted in a "faint band" (Table 2). When adjusted for age and gender, the odds of a ParaCheck Pf^® ^"faint band" test result for those > 5 years as compared to those ≤ 5 years was 0.862 (95% CI = 0.474-1.591, p = 0.607). Only 4.34% (3/69) of "faint bands" corresponded with a true positive, and 94.20% (65/69) of "faint bands" corresponded with true negatives (Table 2). The remaining 1.46% (1/69) of "faint bands" could not classified as there was a disagreement in results between QC readers and therefore it was excluded. When ParaCheck Pf^® ^bands were classified as positive the inter-rater agreement with the gold standard of external QC was 75.62% (kappa = 0.091, SE = 0.0279, p = 0.0006). In contrast, when ParaCheck Pf^® ^test bands were classified as negative the inter-rater agreement with the QC gold standard was 97.53% (kappa = 0.3511, SE = 0.0592, p < 0.001).

**Table 2:** The proportion of faint band appearance, ParaCheck Pf^® ^misdiagnosis with either positive or negative "faint band" classification, local microscopy misdiagnosis and true positives.

ParaCheck^® ^faint bands and misdiagnosis	Proportion
ParaCheck Pf^® ^tests resulting in a faint band (n = 291)	69 (23.7%)

ParaCheck Pf^® ^positive (if classify faint as +) (n = 291)	74 (25.4%)

ParaCheck Pf^® ^positive (if classify faint as -) (n = 291)	5 (1.7%)

ParaCheck Pf^® ^Misdiagnosis (if classify faint as +) (n = 283)	68 (23.4%)

ParaCheck Pf^® ^Misdiagnosis (if classify faint as -) (n = 283)	1 (0.35%)

Misdiagnosis with local microscopy (n = 283)	131 (45.0%)

True positives determined by external QC (n = 283)	6 (2.1%)

ParaCheck Pf^® ^faint Bands corresponding with true positives* (n = 69)	3 (4.3%)

ParaCheck Pf^® ^faint Bands corresponding with true negatives* (n = 69)	65 (94.2%)

*True positives and true negatives were determined by external quality control

### Malaria Misdiagnosis

Misdiagnosis of malaria is defined as any outcome where the local hospital microscopy or ParaCheck Pf^® ^test result did not agree with microscopy QC results. When "faint bands" were classified as positive tests, the proportion of ParaCheck Pf^® ^misdiagnosis was 23.4% (95% CI = 18.5%-28.3%)(Table 2). If "faint bands" were classified as negative, misdiagnosis by ParaCheck Pf^® ^decreased to only 0.353% (95% CI = -0.33%-1.0%) (Table 2). These misdiagnosis proportions are in contrast to the 45.0% (95% CI = 39.3%-50.8%) of patients that were misdiagnosed using local microscopy alone. Results from multivariate logistic regression indicate that the odds of misdiagnosis by local microscopy for those > 5 years as compared to those ≤ 5 years were 0.370 (95% CI = 0.1733-0.7915, p = 0.010). In contrast, even when ParaCheck Pf^® ^faint bands are considered positive, the odds of misdiagnosis by ParaCheck Pf^® ^for those > 5 years as compared to those ≤ 5 years were 0.837 (0.459-1.547, p = 0.5383).

### Sensitivity and Specificity

#### ParaCheck Pf^®^

When ParaCheck Pf^® ^"faint bands" were classified as true positives, specificity was 75.5% (95% CI = 70.3%-80.6%) as compared to 98.9% (95% CI = 97.0%-99.8%) when "faint bands" were classified as negative. For blood slide parasitemia results > 600 parasites/μL (assuming 8,000 leukocytes/μL) the sensitivity was 100% for both the "faint band" negative and positive classifications. When the "faint bands" were classified as negative, 3 out of 69 patients with parasitemia values ≤ 600 parasites/μL were not detected and became false negatives.

#### Microscopy

The sensitivity of blood smears performed by the local hospital was 83.3% (95% CI = 35.9%-99.6%) and specificity was 54.4% (95% CI = 48.4%-60.3%). All values were calculated using the external QC microscopy as the "gold standard".

## Discussion

Prior to the start of our research, the local hospital records estimated the prevalence of malaria in patients presenting with malaria-like symptoms at the out-patient department to be 46% in 2008 [29]. This is significantly higher than the expected prevalence in a low transmission zone. The unusually high reported prevalence sparked our interest into the accuracy of local microscopy and the potential usefulness of RDTs in this area. During this study we confirmed that the actual prevalence of malaria is much lower than previously reported and were able to begin to provide a correction in the understanding of local epidemiology at the hospital.

Further, in this study correct interpretation of ParaCheck Pf "faint bands" as negative was found to be associated with a decrease in misdiagnosis (especially for children under five years) as compared to local microscopy. These results may indicate that the use of RDTs will provide an objective parasitological tool to accurately diagnose malaria in children and adults. As the current WHO Guidelines for malaria diagnosis and treatment still promote presumptive diagnosis of malaria for children < 5 years in areas where parasite-based diagnosis is not available, RDTs may have important implications for reducing over-diagnosis in resource poor areas [30]. Moreover, the objectivity of RDTs may have important implications for: quality of patient care, reducing unnecessary treatment costs, correcting misperceptions of therapeutic failures and ultimately reducing the likelihood of ACT drug resistance in *Plasmodium falciparum *[11,31].

### Impact of incorrectly interpreting "faint bands"

In the current clinical environment where training and experience results in presumptive malaria treatment and belief that "a negative blood slide does not rule out malaria" it is essential for training algorithms to clarify how to interpret "faint bands" occurring in ParaCheck Pf^® ^and potentially other RDTs should be interpreted [3]. Here, we provide compelling evidence that in areas of low transmission, "faint bands" should be considered a negative test when used to inform clinical decision-making. Although this may be at odds with current WHO recommendations (http://www.wpro.who.int/sites/rdt), we assert that this is an important consideration in areas of low transmission.

In this setting, if faint bands are interpreted as true positives and the patient is treated with anti-malarial drugs, this may result in the wrong disease being treated and augment morbidity and unnecessary drug expenses. At the health system level, incorrect interpretation may lead to decreased trust of RDTs, create perceptions of treatment failures and result in an over-estimation of the prevalence of malaria in the area. The consequences are a reinforcement of the culture of presumptive treatment and inaccurate treatment of patients. Especially for areas of low transmission, it is vital to have an accurate prevalence estimate in order to track epidemiological trends and the potential emergence of epidemics due to factors such as climate change.

### Possible reasons for "faint bands"

Although we have not conducted targeted studies to determine the precise underlying reason that "faint bands" appear we propose the following hypothesis based on previous explanations of false positives. ParaCheck Pf^® ^uses a loop applicator with the interior of the loop applicator being calibrated for 5 μl. In field settings the method of blood application is usually dropping the blood onto the loop, leading to the formation of a bubble above the loop structure. The result is the addition of greater than 5 μl to the test strip. Application of too much blood has been identified previously as a contributing factor to false positive results, although the authors did not discuss band intensity [6]. Further, the HRP2 antigen, detected by the ParaCheck Pf^® ^RDT, has been previously shown to remain in the bloodstream up to 60 days following anti-malaria treatment leading to false positive test results [30]. Future research should focus on determining a definitive understanding of what causes "faint bands" in various contexts.

### Potential limitations

It may be possible that "faint bands" are due to low-level parasitemias that are commonly found in areas of low prevalence and often not detectable by microscopy, [1,10], but we anticipate that the examination of blood smears by two highly trained technicians helped to mitigate this risk. For future studies, the gold standard would be to confirm all microscopy results using polymerase chain reaction (PCR). There is also the possibility that patients had HRP-2 remaining in the blood following treatment of a previous case of malaria [2]. We attempted to mitigate this issue by asking patients to disclose previous cases of malaria in the questionnaire and reviewing patient files for recorded previous cases.

Furthermore, it is important to remember that ParaCheck Pf^® ^categorized three patients with parasitemias < 600 parasites/μL as false negatives when "faint bands" were considered negative. The failure to treat these true positive cases will have important ramifications for the individual patient and their families (for example delayed treatment may lead to the development of serious disease and ultimately death of the patient). The risks associated with both over and under treatment of malaria are serious and have been previously discussed [32-34]. So, there remains the critical factor of clinical judgment in the decision to treat or not to treat when the result is a "faint band".

## Conclusions

This research shows that in an area of low malaria transmission, over 94% of "faint bands" are associated with negative microscopy results. Additionally, ParaCheck Pf^®^, when interpreted correctly is a useful tool for accurately estimating prevalence in a defined population and reducing misdiagnosis of children under the age of five in a low transmission setting. Discerning how to interpret "faint bands" and the resulting impacts on malaria misdiagnosis in areas of medium to high transmission and may give different results and therefore we advocate for further research that will add to the growing body of knowledge regarding RDTs and their inherent strengths and limitations.

## Abbreviations

ACT: artemisinin-based combination therapy; BUCHS: Bugando University College of Health Sciences; CHREB: Conjoint Health Ethics Board; COSTECH: Commission on Science and Technology; HRP2: histidine rich protein 2; NIMR: National Institute of Medical Research; NCA: Ngorongoro Conservation Area; QC: Quality Control; RDT: rapid diagnostic test; WHO: World Health Organization.

## Conflict of interest

The authors declare that they have no competing interests.

## Authors' contributions

LKA planned the study, conducted the field research, data analysis and interpretation and wrote the manuscript. JMH contributed to study planning, was involved in the field research and reviewed the manuscript. GD planned the study, conducted field research and reviewed the manuscript. JH was involved in the field research, data analysis and writing of the manuscript. MM contributed to study planning, was involved in field research and reviewed the manuscript. All authors read and approved the final manuscript.

## Pre-publication history

The pre-publication history for this paper can be accessed here:

http://www.biomedcentral.com/1471-2334/11/308/prepub

## Supplementary Material

Additional file 1 **Questionnaire: Malaria and Rapid Diagnostic Tests**. This questionnaire was designed to support the testing of the ParaCheck Pf^® ^through collection of demographic information on study participants as well as investigating their reported malaria health-seeking behaviors. In collaboration with our research personnel, a clinical officer or physician at the local hospital administered either in KiSwahili or Maa the questionnaire to consenting participants. The questionnaire was pilot tested for validity and reliability. During translation the questions were translated into KiSwahili by author MM and then back translated into English by another local research assistant. Click here for file
