# Supplementary material for: Reducing malaria misdiagnosis: the importance of correctly interpreting Paracheck Pf® "faint test bands" in a low transmission area of Tanzania
Source: BMC Infect Dis. 2011 Nov 3;11:308. doi: 10.1186/1471-2334-11-308 (PMC3266231; doi:10.1186/1471-2334-11-308)
Supplement: Additional file 1 — Questionnaire: Malaria and Rapid Diagnostic Tests. This questionnaire was designed to support the testing of the ParaCheck Pf® through collection of demographic information on study participants as well as investigating their reported malaria health-seeking behaviors. In collaboration with our research personnel, a clinical officer or physician at the local hospital administered either in KiSwahili or Maa the questionnaire to consenting participants. The questionnaire was pilot tested for validity and reliability. During translation the questions were translated into KiSwahili by author MM and then back translated into English by another local research assistant. [file 1471-2334-11-308-S1.DOC]

Study #_______________

# Questionnaire – Malaria and RDTs

# This questionnaire is part of the Malaria Rapid Diagnostic Test Study and is designed to gather information regarding malaria health-seeking behaviour of residents in the Ngorongoro Conservation Area, Tanzania. This study is a collaborative research project with Bugando University College of Health Sciences and the University of Calgary, Canada.

| *********PLEASE CHECK THE FOLLOWING ********* | | | | |
| --- | --- | --- | --- | --- |
| **GENDER** | | **AGE** | | |
| MALE | FEMALE | ≤ 5 years | 5 – 18 years | > 18 years |

***Please answer the following questions to the best of your ability.***

1. How long have you been ill with malaria this time?

| <1 day | >1 day | 2 – 7 days | > 7 days |
| --- | --- | --- | --- |

1. How many times have you been ill with malaria in your lifetime?

| 0 – 3 times | 4-7 times | 8 – 11 | 12 or more |
| --- | --- | --- | --- |

1. What was the first thing you did when you thought you were sick with malaria, this time?

­________________________________________________________________________________________________________________________________________________________________________________________________________________________________________________________________________________________________________________________________________

1. Have you seen anyone about treatment for this illness? (i.e. traditional healer, dispensary, elder, other hospital or health centre, family member) Did they give you anything? If so, what did they give you to take?

­________________________________________________________________________________________________________________________________________________________________________________________________________________________________________________________________________________________________________________________________________

1. How long did you walk or travel to get to Endulen Hospital?

­

| 0 – 1 hours | 1 – 2 hours | 2 – 3 hours | > 3 hours |
| --- | --- | --- | --- |

1. Did anyone come with you? (i.e. family members)

­________________________________________________________________________________________________________________________________________________________________________________________________________________________________________________________________________________________________________________________________________

1. Why did you choose to come to the Endulen Hospital?

________________________________________________________________________________________________________________________________________________________________________________________________________________________________________________________________________________________________________________________________________

1. Is there anything else you would like to tell us?

­________________________________________________________________________________________________________________________________________________________________________________________________________________________________________________________________________________________________________________________________________
